# Phosphatidylserine: A Novel Target for Ischemic Stroke Treatment

**DOI:** 10.3390/biom14101293

**Published:** 2024-10-12

**Authors:** Jiaqi Guo, Jiachen He, Shuaili Xu, Xi Chen, Zhanwei Zhu, Xunming Ji, Di Wu

**Affiliations:** 1 Department of Neurology and China-America Institute of Neuroscience, Xuanwu Hospital, Beijing Institute of Brain Disorders, Capital Medical University, Beijing 100053, China; gjq1999@mail.ccum.edu.cn (J.G.); 2022020002@hrbmu.edu.cn (J.H.); chenxi00cx@mail.ccmu.edu.cn (X.C.); sipcc@mail.ccmu.edu.cn (Z.Z.); 2 Beijing Institute of Brain Disorders, Laboratory of Brain Disorders, Ministry of Science and Technology, Collaborative Innovation Center for Brain Disorders, Capital Medical University, Beijing 100069, China; xsl199607@mail.ccmu.edu.cn

**Keywords:** ischemic stroke, phosphatidylserine, multiple targets, neuroprotection

## Abstract

Over the past 40 years, research has heavily emphasized stroke treatments that directly target ischemic cascades after stroke onset. Much attention has focused on studying neuroprotective drugs targeting one aspect of the ischemic cascade. However, the single-target therapeutic approach resulted in minimal clinical benefit and poor outcomes in patients. Considering the ischemic cascade is a multifaceted and complex pathophysiological process with many interrelated pathways, the spotlight is now shifting towards the development of neuroprotective drugs that affect multiple aspects of the ischemic cascade. Phosphatidylserine (PS), known as the “eat-me” signal, is a promising candidate. PS is involved in many pathophysiological changes in the central nervous system after stroke onset, including apoptosis, inflammation, coagulation, and neuronal regeneration. Moreover, PS might also exert various roles in different phases after stroke onset. In this review, we describe the synthesis, regulation, and function of PS under physiological conditions. Furthermore, we also summarize the different roles of PS after stroke onset. More importantly, we also discuss several treatment strategies that target PS. We aim to advocate a novel stroke care strategy by targeting PS through a translational perspective.

## 1. Introduction

Ischemic stroke, caused by an interruption in cerebral blood flow, is one of the leading causes of death and permanent disability worldwide [1,2,3]. Recanalization therapy (intravenous thrombolysis or endovascular mechanical thrombectomy) is currently the most effective treatment for ischemic stroke, but nearly half of patients still have unfavorable outcomes [1,4,5]. The series of pathological and physiological mechanisms due to cerebral ischemia, including excitotoxicity, apoptosis, and inflammation, contribute to poor prognosis after stroke onset [6,7,8]. How to improve the clinical prognosis of patients receiving recanalization therapy is still a major clinical challenge [9]. Therefore, it is an attractive concept to develop neuroprotectants that can interfere with the pathophysiological processes of ischemic stroke. Unfortunately, over the past 40 years, neuroprotectants have focused on targeting one aspect of the pathophysiological processes, which may have a limited impact on the complex pathophysiological mechanisms unleashed by focal cerebral ischemia [10,11]. Hence, new therapeutic strategies affecting multiple aspects of pathophysiological processes for ischemic stroke are increasingly accepted.

The majority of neurons in the ischemic penumbra or peri-infarct zone undergo apoptosis after stroke [12]. The cell membrane phospholipid phosphatidylserine (PS) is a well-known “eat-me” signal [13]. Under physiological conditions, PS is normally located in the inner leaflet of the plasma membrane [14]. However, PS is exposed to the cell surface under pathological conditions, which has long been recognized as a standard marker of apoptotic cells [13]. Interestingly, recent studies have suggested that stressed but viable brain cells after stroke would overexpress PS on the cell surface, especially neurons [15]. The exposed PS on the stressed neurons would lead to engulfment by phagocytes, which would minimize inflammation and reduce neuronal loss in the acute stage [16]. PS exposure would also increase inflammation and exacerbate neuronal loss in the chronic stage [17]. In addition, PS plays a vital role in blood coagulation after stroke. For example, PS exposure on activated platelets promotes the generation of thrombin and fibrin clot formation [18]. A higher PS exposure level was associated with worse neurological function [19]. However, PS might also function as a “save me” signal to participate in neuron regeneration in the process of nervous system recovery [20]. It has been shown that PS can interact with transthyretin (TTR)-like protein 11 to activate the integrin pathway and initiate axonal regeneration [21]. Additionally, exogenous PS treatment had a therapeutic effect on ischemic stroke [22].

Therefore, considering the various roles of PS in apoptosis, coagulation, inflammation, and neuronal regeneration, we hypothesize that PS may be a potential therapeutic target for ischemic stroke. In this review, we describe PS physiological functions under physiological conditions, summarize the pathophysiological role of PS after stroke, and discuss recent advances in stroke therapies targeting PS or administering PS liposomes.

## 2. PS Synthesis, Externalization and Function

### 2.1. PS Synthesis

PS is an essential anionic phospholipid in the cell membrane and plays a pivotal role in membrane structural and functional integrity [23]. In mammals, there are two independent pathways to complete PS biosynthesis [24]. Specifically, phosphatidylserine synthase-1 (PSS1) and -2 (PSS2) are localized in the mitochondria-associated membrane in the endoplasmic reticulum (ER) [25]. PS is also transported from the Golgi to the plasma membrane by traditional vesicle-mediated trafficking [26], which is mainly distributed in the inner leaf of the membrane lipid bilayer to maintain normal cellular function, such as participating in signal transduction, membrane trafficking, and cell survival and proliferation [27,28,29].

### 2.2. PS Externalization

Currently, two key enzymes—flippases and scramblases—have been found to play a crucial role in maintaining PS asymmetry [30]. Flippases, the type IV P-type ATPase (P4-ATPase) family members ATP11A and ATP11C, can transport PS from the extracellular to the cytosolic side with its chaperone Transmembrane protein 30A (TMEM30A). Scramblases, such as Transmembrane protein 16F (TMEM16F) and Xk-related protein (Xkr) 8, transport phospholipids between inner and outer leaflets in a non-specific and bidirectional manner [13]. Under normal physiological conditions, both key enzymes work in coordination to maintain the asymmetric distribution of PS and normal biomembrane physiological function [13]. However, when the cells are subjected to various stimuli, the asymmetry of the membrane lipids is destroyed, resulting in PS eversion.

**Figure 1:**
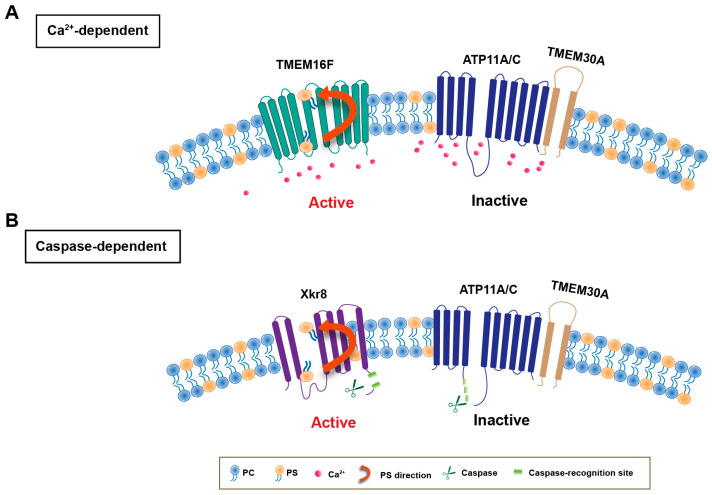
The molecular mechanism for PS exposure. (**A**) Ca^2+^-dependent PS externalization. (**B**) Caspase-dependent PS externalization.

In activated cells or platelets, intracellular Ca^2+^ increases transiently activate TMEM16F to scramble phospholipids and inactivate flippase activity of ATP11A/C [13,31]. When activated cells and platelets return to the normal state, a decrease in intracellular Ca^2+^ inactivates TMEM16F, restores the flippase activity of ATP11C, and re-establishes the asymmetric distribution of PS in the plasma membrane [13]. Put differently, Ca^2+^-induced PS exposure is reversible in activated cells (Figure 1). In contrast to the Ca^2+^-induced PS externalization, it was reported that TMEM16F deficient cells have normal apoptotic PS exposure, suggesting the existence of another independent scramblase. Both Xkr8 scramblase and ATP11A/C flippase have caspase recognition sites [32]. When cells undergo apoptosis, caspase 3 or caspase 7 cleave and deactivate ATP11A/C flippase while simultaneously cleaving Xkr8 to activate its phospholipid scramblase activity, leading to irreversible PS exposure and releasing the “eat me” signal [33]. Then, the macrophages will engulf the apoptotic cells [13,34]. This process is irreversible (Figure 1). Thus, both flippases and scramblases might be upstream targets to regulate PS externalization.

### 2.3. PS Function

**Figure 2:**
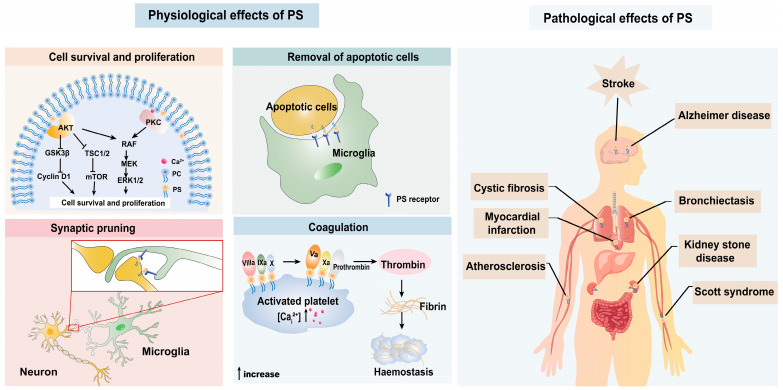
The physiological and pathological effects of PS. Under physiological conditions, PS is involved in cell survival and proliferation, removal of apoptotic cells, synaptic pruning, and coagulation. However, when PS is produced in excess or not removed in time, some diseases, such as cystic fibrosis, Scott syndrome, kidney stone disease, Alzheimer’s disease, and stroke, may happen.

PS, as a protein docking site on the cell membrane, is involved in the activation of various signaling pathways, including protein kinase C (PKC) family, phosphoinositide-3-kinase (PI3K)/protein kinase B (AKT) [23,35]. These signal pathways have been well described to support neuronal cell survival and proliferation [36,37]. Moreover, PS is exposed on the surface of senescent or damaged cells, signaling to macrophages as an “eat me” signal, which allows these cells to be effectively cleared [38]. In activated platelets, PS facilitates the assembly of the tenase and prothrombinase complexes by interacting with the 9-12-carboxyglutamate (Gla) domain of the clotting cascade (FVII, FIX, FX, FII) through Ca^2+^ and promotes the formation of thrombi, playing an important role in blood coagulation [39,40]. Additionally, PS is also involved in microglia-mediated synaptic pruning, which is essential for the assembly of neuronal circuits in brain development (Figure 2) [41,42].

A variety of diseases may occur when PS-exposure cells are produced in excess or not cleared promptly. For example, when clearance is impaired, apoptotic cells due to PS exposure will undergo secondary necrosis, resulting in the release of pro-inflammatory cytokines by phagocytes. These changes will finally lead to cystic fibrosis and bronchiectasis [43,44]. In addition, abnormal distribution of PS will induce Scott syndrome, kidney stone disease, and Alzheimer’s disease [45,46,47]. Moreover, an abnormal increase in PS exposure in platelets or red blood cells causes microvascular occlusion and contributes to thrombosis of arteries and veins, leading to atherosclerosis, myocardial infarction, and other thrombotic diseases [19,48,49]. However, less study makes a summary concerning the effect of PS on ischemic stroke, especially in the era of highly effective reperfusion therapy (Figure 2).

## 3. The Pathophysiology Role of PS Externalization in Ischemic Stroke

### 3.1. Ca^2+^-Dependent PS Externalization in the Acute Stage

Under normal circumstances, the concentration of extracellular Ca^2+^ is about four orders of magnitude higher than that of intracellular Ca^2+^. However, a large amount of Ca^2+^ enters the cytoplasm when cells are exposed to ischemia and hypoxia [50]. In addition, the process of activating scramblase by Ca^2+^ leads to insufficient intracellular energy supply, which inhibits the activity of flippases such as ATP11C and prevents the outer leaflet PS flipped back to the inner leaflet [51].

When SH-SY5Y was exposed to ethanol (EtOH), You and colleagues [52] found an increase in intracellular Ca^2+^ and reactive oxygen species, and PS exposure. Interestingly, when EtOH was removed at the appropriate time, neurons could fully recover to normal levels. This finding indicated that when the cells did not enter the late stage of apoptosis, the damaged neurons could be rescued when neuronal stressors were removed in a timely way. Moreover, Neher et al. [17] found that a low dose of glutamate could induce reversible PS exposure in neurons in vitro acute neuronal injury model. Early PS exposure increased after the glutamate induction, but PS exposure decreased, and neurons resumed normal function after a 24 h recovery. These findings indicated that neurons had the ability to self-recover after mild stress. However, a high dose of glutamate only induced an unreversible PS exposure under the same condition. Thus, the reversibility of neurons under stress might be highly dependent on the degree of damage. This phenomenon was similar to the occurrence and repair process of the penumbra after ischemic stroke.

Animal experiments showed that PS was exposed in the stressed but still viable neurons in the ischemic penumbra after ischemia [15]. In addition, recent studies have demonstrated that TMEM16F, as a Ca^2+^-dependent phospholipid scramblase to regulate PS exposure in living cells reversibly, played a significant role in ischemic stroke [16,53]. It has been reported that in vitro OGD and in vivo MCAO models, knocking down TMEM16F could inhibit the PS exposure of activated neurons and decrease the microglial phagocytosis of viable neurons in the penumbra [16]. More importantly, TMEM16F knockdown could reduce infarct volume and alleviate motor function injury. These findings suggested that regulating neuronal PS exposure may have a neuroprotective effect on ischemic stroke. These findings suggested that TMEM16F knockdown may reduce neuronal apoptosis by regulating PS exposure in a Ca^2+^-dependent way. Taken together, the timely rescue of stressed but surviving neuronal cells in penumbra can have protective effects against ischemic stroke.

### 3.2. Caspase-Dependent PS Externalization in the Sub-Acute Stage

Caspase 3 was a biomarker to differentiate strokes from stroke-mimicking conditions [54]. In oxidatively stressed erythrocytes, caspase 3 activation negatively regulated flippases and led to PS externalization irreversibly, thus increasing phagocytosis of red cells [55]. Inhibition of caspase 3 with Z-DEVD-FMK could block PS exposure and erythrocyte phagocytosis. These findings indicated that caspase 3 activation played a crucial role in inactivating flippase, leading to loss of PS asymmetry and erythrocyte phagocytosis under oxidative stress. Caspase 3 activation depended on the release of cytochrome C [56]. It was reported that neurons that have discharged mitochondrial cytochrome C into the cytoplasm showed no recovery [52]. These studies suggested neurons activated by Caspase 3 were irreparable, which is similar to ischemic core after stroke.

Neher et al. [17] identified that high doses of glutamate would induce irreversible PS exposure in neurons in the neuronal injury model. A higher level of PS exposure was associated with a higher level of caspase 3 and an increased neuronal death. Their findings indicated that neurons cannot self-recover beyond a certain range. During the super-acute phase after stroke, the astrocytes and neurons in the ischemic core were induced to cell death, which was characterized by PS exposure [57]. Subsequently, these dead cells released the damage-associated molecular patterns (DAMP), which further promoted the death of neurons and glial cells in the penumbra. Nakahashi-Oda et al. [58] discovered that enhancing deceased cells’ efferocytosis could attenuate the inflammatory response, mitigate the loss of surviving neurons, and ultimately confer neuroprotective effects.

### 3.3. PS Externalization in the Chronic Stage

In contrast to findings of PS externalization in the sub-acute stage, PS externalization led to different outcomes in the chronic stage. In the chronic stage of stroke, cerebral ischemia was accompanied by a strong inflammatory response, which led to PS externalization on stressed but survived neurons [17,59].

The milk fat globule EGF-like factor-8 (MFG-E8) or Mer receptor tyrosine kinase (MerTK) on microglia would specifically bind to PS on the surface of apoptotic cells and mediate the clearance of these damaged cells by phagocytes [60,61]. Neher et al. [17] found that these two phagocytic proteins were strongly and transiently expressed in the infarct at 3–7 d after cerebral ischemia. Brain macrophages would phagocytose these still viable and functional neurons that expressed PS, leading to brain atrophy and motor dysfunction in the chronic stage of ischemic stroke. Thus, these PS-expressing neurons should be timely protected to minimize neuron loss.

### 3.4. PS Externalization in Platelet

Platelet activation was induced by the translocation of the PS from the inner to the outer leaflet of the plasma membrane bilayer, resulting in assembly and catalysis of intrinsic tenase (factors VIIIa/IXa), prothrombinase (factors Va/Xa), and XIa complexes, leading to thrombin generation and consolidation of the fibrin-platelet plug [62,63]. As reported [31], Ca^2+^ ionophore A23187-stimulated platelets induced intracellular Ca^2+^ increase and rapid PS exposure, thereby increasing procoagulant activity, which was not affected by caspase inhibitor Q-VDOph or Bak^−/−^Bak^−/−^ *mice*. The finding indicated that PS exposure activated platelets through a Ca^2+^-dependent manner while was not dependent on the apoptotic events. Ma et al. [64] found that platelets in sepsis patients showed greater expression of PS than in healthy controls, and platelet–leukocyte complexes were significantly elevated. In in vitro experiments, endothelial cells could activate platelets by binding PS with α V β 3 integrins in sepsis patients, correspondingly reducing activated platelet aggregation and the formation of platelet-leukocyte complexes and significantly reducing the formation of procoagulant enzyme complexes. When PS was blocked with lactadherin or Annexin A5, procoagulant activity on activated platelets was significantly decreased. These findings indicated that PS exposure was directly related to procoagulant activity on activated platelets and played a critical role in platelet removal by endothelial cells. In fact, Yao et al. [19] found PS exposure in the blood cells of ischemic stroke patients. Zhao et al. [65] suggested that in patients with internal carotid artery (ICA) stenosis who have undergone carotid artery stenting (CAS), PS^+^ platelets peaked at 2 h after CAS, and PS^+^ neutrophils and erythrocytes peaked at 48 h after CAS. In addition, PS^+^ blood cell levels were associated with reduced clotting time and significantly increased intrinsic and exogenous Xase, thrombin production, and fibrin formation, indicating that Ca^2+^-induced PS exposure on activated platelets is involved in the generation of thrombin and fibrin in ischemic stroke.

**Figure 3:**
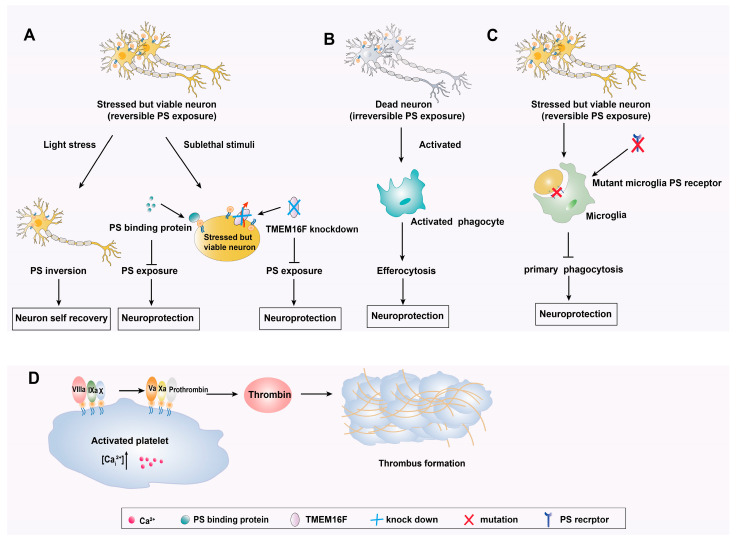
The Pathophysiology role of PS externalization in ischemic stroke. (**A**) When the neurons underwent light stress, internal cascades of survival signaling became triggered to protect against cell death. Neurons had the ability to self-recover after light stress. However, in the subacute phase of ischemia, stressed but survived neurons in the penumbra displayed PS signal to induce microglia phagocytosis, resulting in loss of neurons. These neurons should be saved in time by reducing PS exposure or blocking PS exposure. (**B**) The remaining neurons were induced to die directly due to ischemia and hypoxia, which are characterized by exposure to PS in a caspase-dependent manner. These dead neurons need to be cleared in a timely manner to halt the inflammatory response. (**C**) During the chronic phase, stressed neurons to PS exposure were correlated with delayed neuronal loss. Research has indicated that blocking specific phagocytic pathways can prevent delayed neuronal death and functional impairment. (**D**) Activated platelets after ischemic stroke recruited clotting factors and promoted thrombosis.

In conclusion, targeting PS exposure might have different effects at various time points after stroke onset. In the acute phase, when Ca^2+^-dependent PS exposure was observed in neurons, timely protection and terminating ischemia might lead to a reversible PS exposure and neuroprotection. However, in the subacute phase, when caspase-dependent PS eversion was observed, early clearance of these cells would lead to better neuroprotection. Moreover, in the chronic phase of stroke, reducing PS exposure on neurons might play a neuroprotective role in ischemic stroke. Moreover, PS was also involved in the formation of stroke thrombi, which may be a potential biomarker and drug target for stroke therapy (Figure 3).

## 4. Targeting PS for Ischemic Stroke Treatment

### 4.1. Decreasing PS Exposure Leads to Less Apoptosis

Ischemic stroke induces many pathological changes but ultimately leads to neuron death. Unlike the ischemic core (unsalvageable tissues), where neurons are predominantly necrotic, neurons in the ischemic penumbra (salvageable tissues) are predominantly apoptotic [12]. Apoptotic cells will expose PS, an “eat me” signal, for clearance. Therefore, the strategy of reducing PS exposure in the ischemic penumbra may reduce neuronal apoptosis and improve the prognosis of ischemic stroke. When the cells underwent light stress, internal cascades of survival signaling became triggered to protect against cell death and defend against future insults [66].

However, in the subacute phase of ischemia, these neurons were induced to die directly due to ischemia and hypoxia, which should be cleared in time. Nakahashi-Oda et al. [58] discovered that promoting efferocytosis through the CD300b pathway enhanced phagocyte clearance of PS-positive neurons and reduced inflammatory response. This approach reduced neuron loss in the penumbral region, rescued the neuronal deficit, and eventually played a neuroprotective role. Treatment with an anti-CD300a neutralizing antibody could also improve the neurological deficits after MCAO. Together, timely removal of dead cells (irreversible PS exposure), would protect neurons in the penumbra neurons after stroke onset. In the chronic stage of stroke, specific knockout of MerTK or MFG-E8 would reduce neuronal loss and improve neural function recovery. More importantly, brain atrophy was significantly improved in a mouse MCAO model [17].

Annexin A5 is a Ca^2+^-dependent phospholipid-binding protein with a high affinity for PS [67]. When lipopolysaccharide (LPS) induced injury to the neurons/microglia cultures in vitro, both PS exposure in neurons and the phagocytosis of microglia were increased. PS-binding proteins, such as Annexin A5 or anti-PS antibodies, can significantly decrease PS exposure, reduce the phagocytosis of microglia, and promote neuron survival [68]. This evidence illustrated that decreasing PS exposure might be a potentially valuable therapeutic strategy to inhibit apoptosis for ischemic stroke (Table 1).

### 4.2. Decreasing PS Exposure Also Has an Antithrombosis Effect

Activated platelets were one of the indispensable pathological factors in cerebral thrombosis [76]. Engelke et al. [70] showed that activated FVIIIa would bind to PS-exposing platelets and promote a coagulation cascade. The addition of Annexin A5 could shield FVIIIa from binding to platelets and inhibit coagulation. Additionally, Kuijpers et al. [77] found that PS-exposing platelets were present in a mouse model of arteriolar and venular thrombus development. Arteriole and venule thrombus formation was eliminated by injecting Annexin A5 to shield exposed PS, indicating that PS exposure on activated platelets is a critical factor in thrombus growth in arterioles and venules. However, masking PS with Annexin A5 attenuates thrombosis, but Annexin A5 has a short half-life and is easily cleared by circulation [78]. With a prolonged half-life, Rand et al. [69] found that Diannexin, an Annexin A5 homodimer, reduces the production of platelet–fibrin clots and thrombin in vitro. It also lessened platelet activation and accumulation in *mice* that had experimentally produced arterial thrombi. Moreover, Chen et al. [71] designed four Annexin A5-Kunitz type protease inhibitor fusion proteins, targeting PS-rich thrombogenic sites, which have been proven to effectively suppress thrombogenesis by facilitating the inhibition of the membrane-associated coagulation complexes (TF/FVIIa/FXa/thrombin). Targeting PS-rich thrombogenic locations, four Annexin A5-Kunitz type protease inhibitor fusion proteins have been shown to efficiently prevent thrombogenesis by promoting the inhibition of membrane-associated coagulation complexes. In myocardial ischemia-reperfusion injury *rat*, anticoagulant Annexin A5-6L15 could reduce infarct size with less risk for bleeding side effect by binding to PS on the membrane surfaces of apoptotic cells and inhibiting the membrane-anchored TF/FVIIa [49]. R5421 (ethaninidothioic acid) was previously shown to reduce Ca^2+^-induced PS exposure in activated platelets [79]. Recently, Millington-Burgess et al. [72] reported that R5421 inhibited human platelet PS exposure by maintaining the activity of a flippase protein, thereby reducing thrombosis. Overall, the above research suggested that the exposure of PS from activated platelets and the enzymes that regulated PS exposure may provide an antiplatelet and anticoagulation novel target, and further research is needed to identify its role in ischemic stroke (Table 1).

### 4.3. Increasing PS Exposure Promoting Neuronal Regeneration

After a stroke, axonal fusion is a highly effective way to repair damaged neurons functionally. All that is needed for the regrowing axon to do is cross the injury location and merge with its separated counterpart to restore neuronal function [20]. Recently, it has been found that the damaged axon in the *C. elegans* nervous system exposed PS, which serves as a “save me” signal for the proximal fragment to recognize [80]. This specific recognition of the distal fragment by the proximal fragment promoted axon reconnection and fusion and re-established axonal integrity. Ho et al. [81] reported that PS exposure on the injury axon can define the level of axonal fusion. The degree of axonal fusion is closely correlated with the number of PS exposed to the axonal membrane. Moreover, Abay et al. [82] demonstrated that axonal fusion can restore complete function to damaged neurons and drive rapid functional recovery of the nervous system.

Mechanistically, Noumann et al. [80] used a candidate gene screening method to identify molecules involved in recognition between regenerated axons and their separated segment used a candidate gene screening approach. Surprisingly, axonal fusion shares molecular pathways with the recognition and phagocytosis of apoptotic cells. In one of the redundant engulfment pathways, the PS receptor (PSR-1) is involved in axonal fusion [80]. Hisamoto et al. [21] proposed an axonal fusion mechanism in which *CED-7* and *TTR-52* promote the release of PS from the distal segment to the regenerating axonal segment after it has been exposed in the context of axonal injury. TTR-11 protein activates integrin by binding to the extracellular domain of integrin-α subunit INA-1 and PS. The signal is transmitted within the cell through the *CED-2-CED-5-CED-12* module and *CED-10* to promote axonal fusion. Ho et al. [81] demonstrate that the role of ADM-4 metalloprotease in the setting of axonal fusion depends on its activity and its binding to PS. Together, these findings provided hope for future studies on stroke recovery by indicating that axonal fusion, which recovered damaged neurons to full function, depended on exposure to PS signals.

### 4.4. PS as a Drug Delivery Vehicle for Ischemic Stroke Treatment

Currently, targeting microglial activation is a potential cytoprotective strategy in acute ischemic stroke. Recent studies have found that PS has the potential as a drug delivery vehicle to help astrocytes and microglia drug uptake, thus exerting an anti-inflammatory effect [83]. Due to the crucial role of PS in apoptosis, PS liposomes (PSL) have been used as a strategy to mimic the apoptotic cells in terms of PS presentation on their surface and their anti-inflammatory effects [84]. Partoazar et al. [73] identified that in a permanent cerebral ischemia *mice* model, oral PS liposomes significantly reduced the levels of TNFα in the brain and survived hippocampal neurons after brain ischemia and reduced infarct brain. Their findings indicated that PS liposomes may have a dual role, including inflammation control and neural proliferation in brain compartments like the hippocampus. Moreover, Zhao et al. [74] fabricated PS-modified microbubbles, which could cross the blood-brain barrier (BBB) and target the activated microglia/macrophages. Their findings indicated that in an ischemic stroke *rat* model, PS-modified microbubbles could improve BBB permeability and modulate microglial activation toward an anti-inflammatory phenotype, which achieved novel delivery routes for targeting the inflammation area. In addition, Bai et al. [75] developed PS and transferrin (TF) modified liposomes carrying Danshensu (DSS) (TF/PS/DSS-LPs). They identified that PS modification enabled liposomes to target and bind to astrocytes and microglia, which overexpressed PS-specific receptors in ischemic stroke. The TF/PS/DSS-LPs can inhibit astrocytic and microglial activation through NF-κB p65 and TLR4 in ischemic injury, thus reducing neuronal inflammation and ultimately ameliorating cerebral ischemic injury. Together, PSL, as a drug carrier, is promising and potential for novel and effective treatments for ischemic stroke (Table 1).

## 5. Future Perspectives

As discussed in earlier sections, two mechanisms of PS externalization (Ca^2+^-dependent and Caspase-dependent) exist for regulating the asymmetric distribution of PS, which had varying effects in different periods of stroke. During a super acute stroke, a large number of dead neurons exposed PS. Damaged neurons should be cleared in time by promoting efferocytosis, thereby reducing inflammation and ultimately playing a neuroprotective role. However, in the chronic phase, inhibition of phagocyte clearance of damaged neurons can prevent the delayed loss of neurons and thus play a neuroprotective role. The above studies only focused on the phagocytosis of “PS exposure” neurons in hyperacute and chronic stages and did not evaluate the relationship between direct regulation of “PS exposure” and stroke prognosis. Whether directly reducing PS eversion level has a neuroprotective effect needs further exploration during the hyperacute or chronic phase of stroke.

Currently, the combination of reperfusion therapy and cytoprotection drugs seems to be an effective treatment for ischemic stroke. In a recent clinical trial, the cytoprotection drug nerinetide in combination with thrombectomy seems to have good therapeutic benefits in some patients, providing new hope [85]. The findings of the ESCAPE-NA1 trial of nerinetide provide an important idea that we need to develop neuroprotective drugs that affect multiple aspects of the ischemic cascade. Due to its involvement in apoptosis, inflammation, and coagulation during ischemia-reperfusion, PS may indicate a tractable target in ischemic stroke. However, the specific biological effects of PS in ischemic stroke are still largely unknown and warrant further research.

PS has been used directly as an oral drug over the past few decades. In Alzheimer’s disease patients, oral PS could significantly improve cognitive function and memory loss based on the previous findings that microglia secreted prostaglandin E2 (PGE2), a potent anti-inflammatory molecule in the central nervous system [86]. In a double-blind, randomized controlled study, PS therapy improved memory scores when compared to a placebo in elderly Japanese subjects with memory complaints [87]. PS supplementation also exerted benefits in children with attention-deficit/hyperactivity disorder (ADHD) [88]. A meta-analysis suggested that PS therapy of 200–300 mg/day may be effective in reducing symptoms of inattention in children with ADHD [89]. Moreover, a preliminary study found that PS (100 mg) may have a positive impact on cognitive function in older people (*n* = 30) with memory impairment over a period of 12 weeks [90]. However, in another double-blind study, PS therapy of 300–600 mg/day failed to lead to any significant improvement in cognitive skills in 81 subjects with age-related memory impairment (AAMI) for 12 weeks [91]. These studies can only serve as preliminary low-quality evidence. Thus, it is still uncertain whether oral PS could result in better clinical outcomes. Further rigorous clinical studies are still needed.

PS can also serve as a drug delivery tool to elevate the bioavailability of drugs. In an ischemic stroke *rat* model, PS-modified microbubbles could cross the BBB and target the activated microglia/macrophages [74]. Moreover, PS liposome treatment promoted the production of anti-inflammatory factors and inhibited the production of pro-inflammatory in phagocytes [22,73,92]. These findings suggested that PS-containing cargos possessed a capacity to treat ischemic stroke and provided new directions for future stroke research.

In addition, the neuroprotective effect of targeting PS has been widely recognized in preclinical models, but its clinical application has not been sufficiently developed due to the limitations of its detection technology. Recently, some newly developed methods for PS detection have the potential to be translated into clinical trials. Advances in the development of Magnetic resonance imaging have allowed the non-invasive detection of PS exposure in tumor cells in vivo, which can evaluate the early response of tumors to treatment [93]. This would facilitate the detection of PS exposure in stroke patients.

Hence, further studies on PS exposure will be necessary to explore their potential as an early biomarker and therapeutic target in ischemic stroke. Together, further research is needed to identify the exact role of PS in ischemic stroke.

## 6. Conclusions

This review provides an overview of recent findings on PS under physiological and pathophysiological conditions, aiming to elucidate the potential of PS-targeting stroke management from a translational perspective. Of note, these findings have substantial implications for studying multi-target therapy. As a consequence, PS is of great significance as a potential target for multi-target therapy in ischemic stroke, and PS-containing cargos possess a capacity to treat ischemic stroke, providing directions for future research.

## Figures and Tables

**Table 1:** Therapy for targeting PS or administering PS liposome-related pathophysiology in ischemic stroke.

Targeting Pathophysiology	Effect/Function of PS	Therapy	Applications	References
Apoptosis	A functional ligand for CD300a	PS receptor CD300a deficient or anti-CD300a neutralizing antibody	*Mice* MCAO	[58]
	Promoting microglia phagocytosis	PS receptor MerTK Bridging molecule MFGE8 mutation	*Rat* MCAO/ In vitro	[17]
	Promoting microglia phagocytosis	Anti-PS antibody or Annexin A5	In vitro	[68]
Coagulation	Exposure to activated platelets promotes thrombosis	Diannexin or Annexin A5	In vitro	[69]
	Binding to activated FVIIIa promotes a coagulation cascade	Annexin A5	In vitro	[70]
	Promoting the assembly of coagulation complexes	Annexin A5-Kunitz type protease inhibitor fusion proteins	*Rat*/In vitro	[71]
Antithrombosis	Maintaining flippase activity in procoagulant platelets	R5421	In vitro	[72]
Inflammation	Inhibit inflammation	PS liposome	*Mice* MCAO	[73]
	Target activated microglia for injury area	PS-modified microbubbles	*Rat* MCAO	[74]
	Target activated microglia and astrocytes for injury area	TF/PS/DSSLPs	*Rat* MCAO/ In vitro	[75]
